# Signaling Pathways Governing the Caspofungin Paradoxical Effect in Aspergillus fumigatus

**DOI:** 10.1128/mBio.01816-20

**Published:** 2020-08-18

**Authors:** Nicolas Papon, Florent Morio, Dominique Sanglard

**Affiliations:** a Host-Pathogen Interaction Study Group (GEIHP, EA 3142), UNIV Angers, Angers, France; b Federative Structure of Research Cellular Interactions and Therapeutic Applications, SFR 4208 ICAT, Univ Angers, Angers, France; c Laboratoire de Parasitologie-Mycologie, Institut de Biologie, CHU Nantes, Nantes, France; d Département de Parasitologie Et Mycologie Médicale, EA1155 IICiMed, Institut de Recherche en Santé 2, Université de Nantes, Nantes, France; e Institute of Microbiology, Department of Laboratory, Lausanne University Hospital, Lausanne, Switzerland

**Keywords:** *Aspergillus fumigatus*, caspofungin, tolerance, paradoxical growth, transcription factors

## Abstract

Aspergillus fumigatus is responsible for a wide range of diseases affecting several million people worldwide. Currently, a few families of antifungals are available to fight aspergillosis, and we are facing a worrisome increase in resistance to azoles, the drugs used for both first-line treatment and prophylaxis of invasive aspergillosis. In this context, some of the latest antifungals, i.e., echinocandins, have gained attention. Even though acquired resistance to echinocandins is yet uncommon in A. fumigatus clinical isolates, some strains exhibit another characteristic that relies on their capacity to grow at suprainhibitory echinocandin concentrations *in vitro*. This intriguing phenomenon, especially observed with caspofungin and now referred to as the caspofungin paradoxical effect (CPE), relies on molecular mechanisms that were hitherto little understood. Here, we discuss the recent key findings of Valero and colleagues published in *mBio* (C. Valero, A. C. Colabardini, J. Chiaratto, L. Pardeshi, et al., mBio 11:e00816-20, 2020, https://doi.org/10.1128/mBio.00816-20) that will allow a better understanding of the complex regulatory pathway involved in governing the response of A. fumigatus to caspofungin.

## Abstract (precis)

Aspergillus fumigatus is responsible for a wide range of diseases affecting several million people worldwide. Currently, a few families of antifungals are available to fight aspergillosis, and we are facing a worrisome increase in resistance to azoles, the drugs used for both first-line treatment and prophylaxis of invasive aspergillosis. In this context, some of the latest antifungals, i.e., echinocandins, have gained attention. Even though acquired resistance to echinocandins is yet uncommon in A. fumigatus clinical isolates, some strains exhibit another characteristic that relies on their capacity to grow at suprainhibitory echinocandin concentrations *in vitro*.

## COMMENTARY

Fungal infections represent a major problem in human health as viral or bacterial infections (1). Of these, invasive infections caused by the mold Aspergillus fumigatus which occur in immunocompromised patients, and especially neutropenic or solid-organ transplant recipients, are responsible for up to 100,000 deaths a year worldwide (2).

Therapeutic options are still based on a few families of antifungals. First-line therapies vary according to local recommendations, diseases, and host factors. Currently used antifungals include the triazoles, amphotericin B, and the echinocandins (caspofungin, micafungin, and anidulafungin). However, we are facing a worrisome increase in azole resistance due to both long-term azole therapy and environmental practices. As a matter of fact, azole fungicides are being used in agriculture for crop protection and are known to cause cross-resistance with medical azoles (3). Hence, developing innovative therapeutic strategies is essential to better fight invasive aspergillosis. In this regard, many hopes are founded today on the echinocandins, the latest family of antifungals. The potential of these semisynthetic molecules relies on three main characteristics: (i) their target (β-1,3-glucan synthase) being fungus specific and different from that of the other systemic antifungals, thus opening synergistic potential with other classes; (ii) their efficiency at low concentrations; and (iii) a low toxicity. As for *Candida* species, echinocandin resistance in A. fumigatus is mainly due to mutations in the *fksA* gene encoding the target of echinocandins, i.e., the A subunit of the multimeric 1,3-β-d-glucan synthase, but seems rather uncommon (4).

Beyond acquired resistance toward echinocandins, another characteristic is the capacity of some A. fumigatus strains to still grow at suprainhibitory echinocandin concentrations *in vitro*. This intriguing phenomenon, formerly described with the echinocandin cilofungin in the opportunistic yeasts Candida albicans and Candida tropicalis, was referred to as the paradoxical effect (5). The paradoxical effect could be also understood as a result of drug tolerance, which allows survival and/or sustained residual growth at drug concentrations above the MIC. Intense research conducted in *Candida* spp. has led to the conclusions that the paradoxical effect is strain, species, and echinocandin molecule dependent (5). In such a perspective, some molecular determinants behind the caspofungin paradoxical effect (CPE) or caspofungin tolerance were characterized in recent years. These include stress response pathways (Hsp90, calcineurin, mitogen-activated protein kinases), reactivation of β-1,3-glucan synthase, and aneuploidy modulating calcium metabolism and remodeling of the cell wall. Although experimental data support its existence *in vivo*, there is yet no evidence for the relevance of CPE when treating invasive candidiasis with echinocandins. By comparison, so far less is known in A. fumigatus, but this topic has recently gained attention given the possibility to treat invasive aspergillosis with echinocandins (6).

A few years ago, Loiko and Wagener demonstrated that CPE could be explained by the recovery of β-1,3-glucan synthase activity (7). In a preliminary work, Goldman and colleagues demonstrated the role of the basic leucine zipper ZipD transcription factor (TF) in regulating the calcium-calcineurin pathway that governs CPE in A. fumigatus (8). To gain further insights into the regulation of the CPE, and considering that this phenomenon could result from the concerted effect of many gene regulators, these authors took the opportunity of a 484-TF null mutant deletion library to address this question (9, 10).

**FIG 1:**
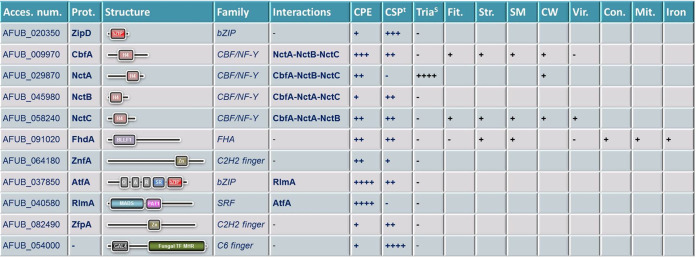
Characteristics of TFs identified in this study and summary of phenotypes observed for each corresponding gene mutant. Abbreviations: Acces. num., accession number in NCBI; Prot., name of the corresponding predicted protein; CPE, caspofungin paradoxical effect; CSP^t^, caspofungin tolerance; Tria^S^, triazole susceptibility; Fit., fitness defect of the strain; Str., involvement in stress adaptation; SM, involvement in regulation of secondary metabolism; CW, role in cell wall remodeling; Vir., role in virulence; Con., involvement in conidiation; Mit., involvement in mitochondrial function; Iron, involvement in iron uptake and metabolism; bZIP, basic leucine zipper domain; H4, histone H4 domain; BLLF1, herpes virus major outer envelope glycoprotein; Zn, zinc finger—C_2_H_2_ type; O, Aft1 osmotic stress response (OSM) domain; A, Aft1 HRA domain; R, Aft1 HRR domain; SR, serine-rich region of AP3B1, clathrin-adaptor complex; MADS, MCM1, Agamous, Deficiens, and SRF (serum response factor) box family; PAT1, topoisomerase II-associated protein; GAL4, GAL4-like Zn(II)2Cys6 (or C6 zinc) binuclear cluster DNA-binding domain; Fungal_TF_MHR, fungal transcription factor regulatory middle homology region; CBF/NF-Y, C-repeat binding factor/nuclear factor Y family; FHA, forkhead-associated domain; SRF, serum response factor; +, weak implication; ++, moderate implication; +++, high implication; ++++, strong implication; −, no implication.

By testing this mutant library for *in vitro* susceptibility to high concentrations of caspofungin, they first identified a set of 11 TFs controlling the CPE (Fig. 1). Importantly, all but two were demonstrated to be important not only for CPE but also for caspofungin basal susceptibility (10).

Four of these TFs, named NctA, NctB, NctC, and CbfA, are related to the C-repeat binding factor/nuclear factor Y family (CBF/NF-Y), which regulates the expression of nuclear genes that function in eukaryotes’ mitochondrial respiration (11). Interestingly, functional gene network predictions suggested that these four TFs are able to interact together under conditions leading to caspofungin tolerance.

By conducting more detailed phenotype analysis in some of these TF mutant strains, Valero and colleagues (10) nicely demonstrated that NctC and CbfA are also essential for optimal growth under various stress conditions. Unexpectedly, both CBF/NF-Y TFs were also shown to act as negative regulators of the biosynthesis of secondary metabolites belonging to the nonribosomal peptide family (NRPs) such as fumiquinazolines A, C, and F; pyripyropene A; tryprostatin A; and fumitremorgin C. Last but not least, the *nctC* null mutant was shown to be avirulent in a neutropenic mouse model. Taken together, besides CPE, these data provide evidence for the multiple functions and complex interplay of NctC and CbfA in the coordinated regulation of various gene clusters for specialized metabolite biosynthesis and virulence.

Interestingly, another previously uncharacterized TF (FhdA) that encodes a forkhead-associated domain was first shown as important for both caspofungin and cell wall stress tolerance. In addition, transcriptomic analysis revealed that the *fhdA* null mutant displayed marked defects in the mitochondrial function that likely influence cellular pathways regulating caspofungin tolerance. Importantly, as for NctC and CbfA, transcriptome sequencing (RNA-seq) data also revealed a potential role of FhdA in the regulation of secondary metabolite biosynthesis. Further investigations provided unprecedented insight into the central role of iron homeostasis in modulating caspofungin sensitivity and CPE through FhdA. Indeed, when *fhdA* mutant cells were exposed to caspofungin, the authors noticed the induction of several genes involved in iron metabolism, particularly those participating in secondary metabolite-type siderophore biosynthesis. The biological relevance of this observation is indeed provided: iron depletion from the medium increased caspofungin sensitivity and decreased CPE in the *fhdA* mutant, while supplementation of the medium with iron promoted CPE. Taken together, these results link iron homeostasis with echinocandin tolerance.

In conclusion, this excellent piece of work highlighted novel mechanistic insights into a complex network of TFs controlling basal caspofungin susceptibility and CPE in A. fumigatus. It also provides evidence that echinocandin tolerance likely relies on the orchestration of multiple cellular processes ranging from cell wall remodeling and calcium or iron metabolism to mitochondrial respiratory function. Therefore, these cellular pathways acting during caspofungin responses could be considered in the near future as innovative fungal targets for therapeutic development.

Beyond these speculative considerations, this work is another proof of the added value of mutant library screens that has long been hampered in A. fumigatus, due to the difficulty of engineering this species. However, thanks to collaborative efforts, Furukawa et al. recently reported the first large A. fumigatus deletion library of TFs that allowed the identification of 12 TFs involved in itraconazole susceptibility (9). The study by Valero et al. is another application of this library and illustrates how genetic manipulation will allow streamlining research in medical mycology in order to decipher the genetic basis of drug resistance and virulence in this dreadful mold.

Finally, this enlightening article provides evidence that cell signaling circuitries modulating caspofungin response in A. fumigatus concomitantly engaged the regulation of various secondary metabolism pathways. Given that commercially available echinocandins rely on a natural product (pneumocandin B_0_) from the Ascomycetes mold Glarea lozoyensis, the global regulation of the secondary metabolism observed in A. fumigatus when exposed to caspofungin could thus reflect basic processes engaged in the interspecies chemical warfare occurring in nature (12).
